# Supplementary material for: Exploring the effects of exercise on immune cell function and tumour infiltration in patients with breast cancer receiving neoadjuvant chemotherapy – a feasibility trial
Source: Brain Behav Immun Health. 2025 May 20;46:101021. doi: 10.1016/j.bbih.2025.101021 (PMC12159211; doi:10.1016/j.bbih.2025.101021)
Supplement: Multimedia component 1 [file mmc1.pdf]

Panel 1

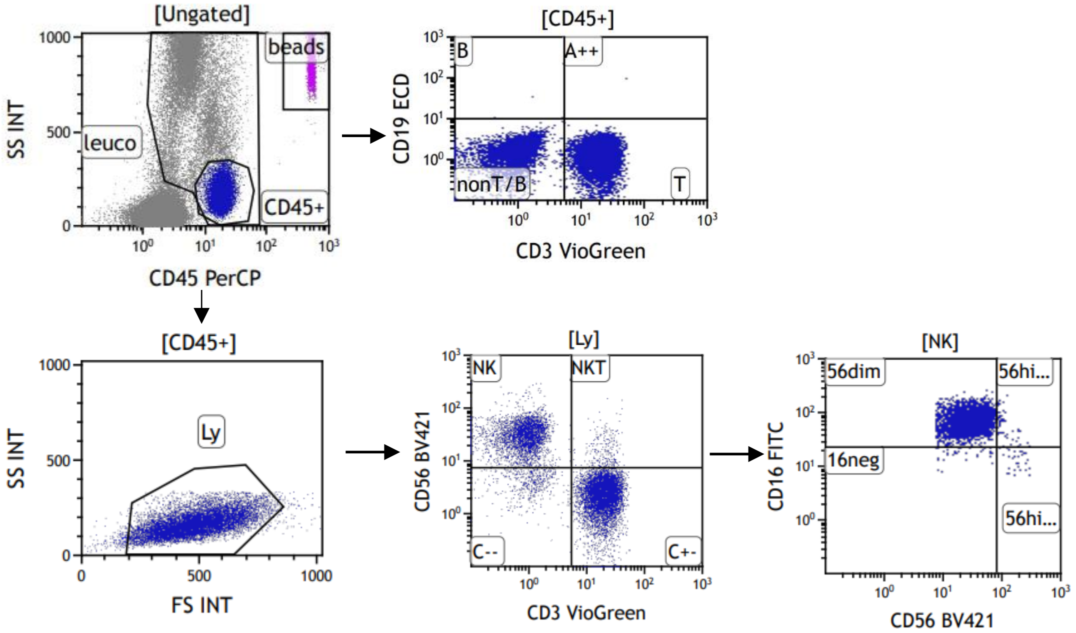

Panel 2

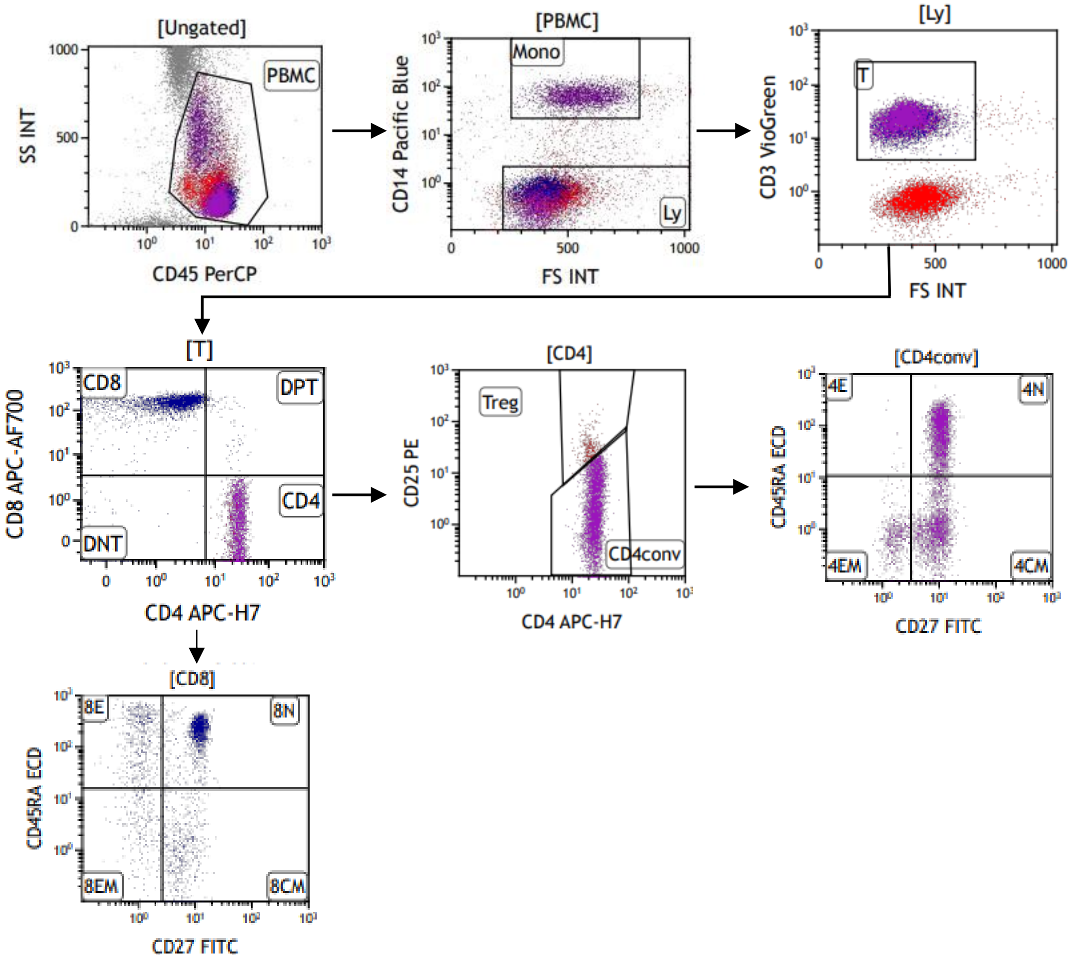

Supplementary Figure 1. Gating strategy immune cell profiling.

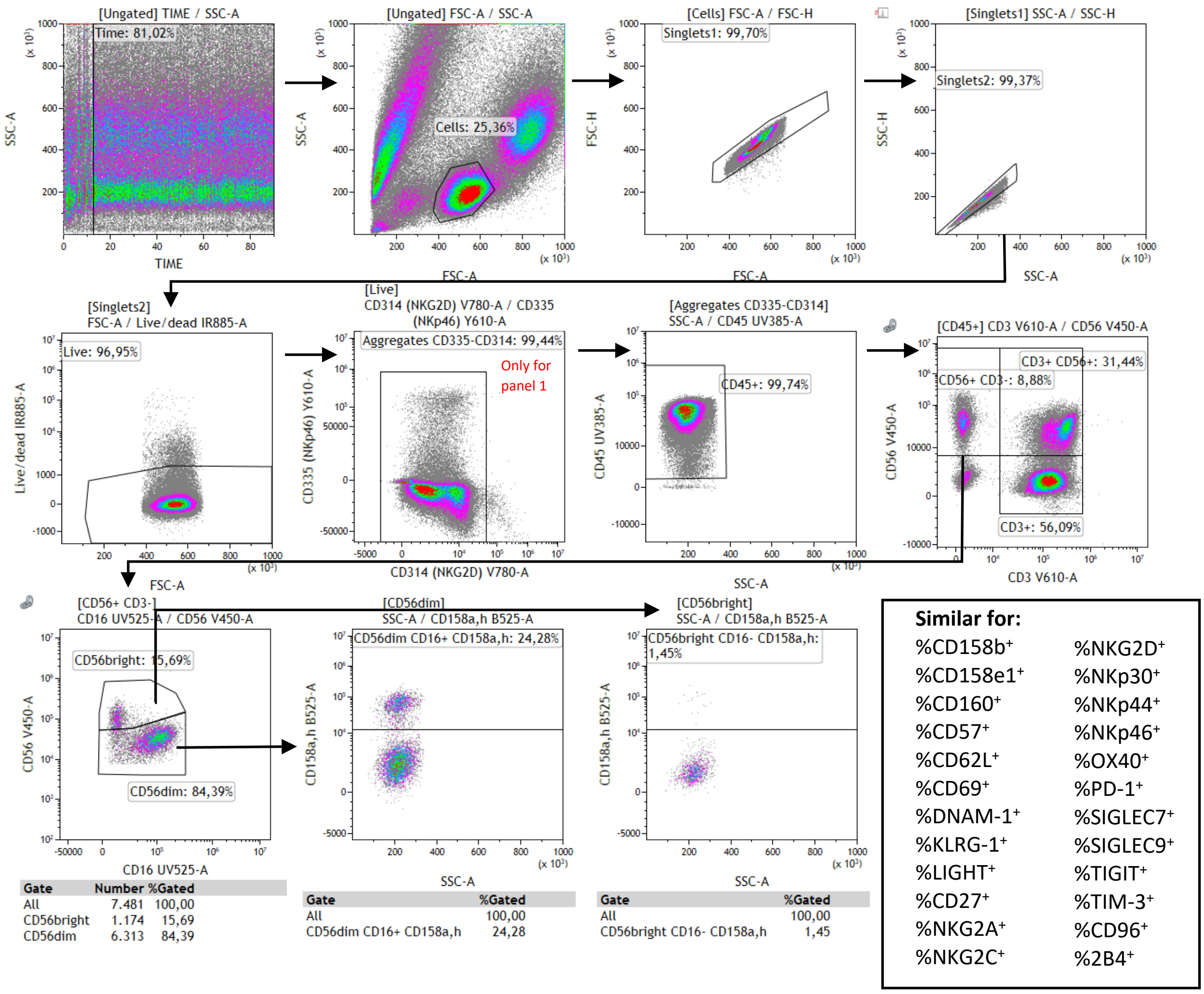

Supplementary Figure 2. Gating strategy NK cell phenotype.

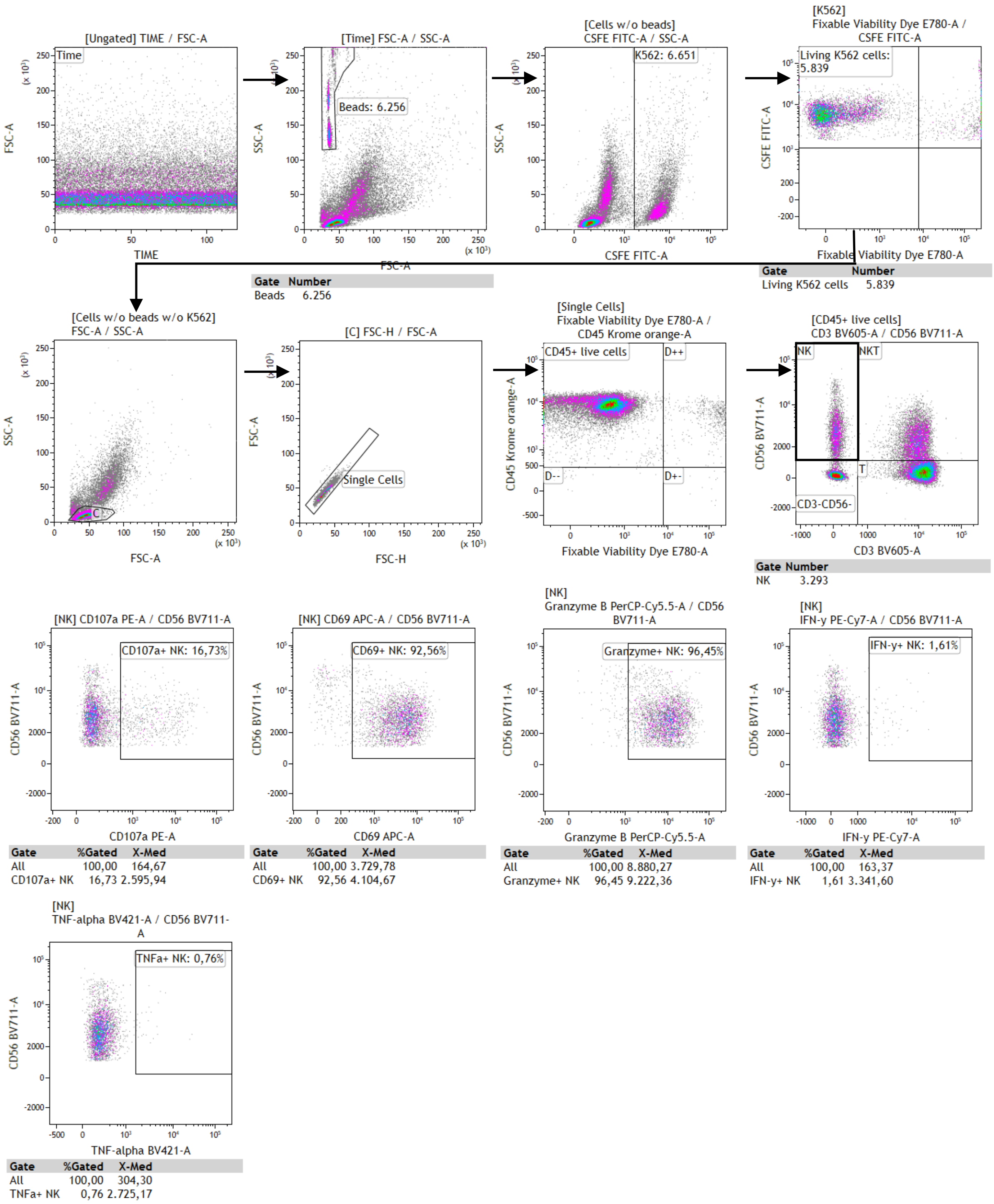

Supplementary Figure 3. Gating strategy NK cell function.

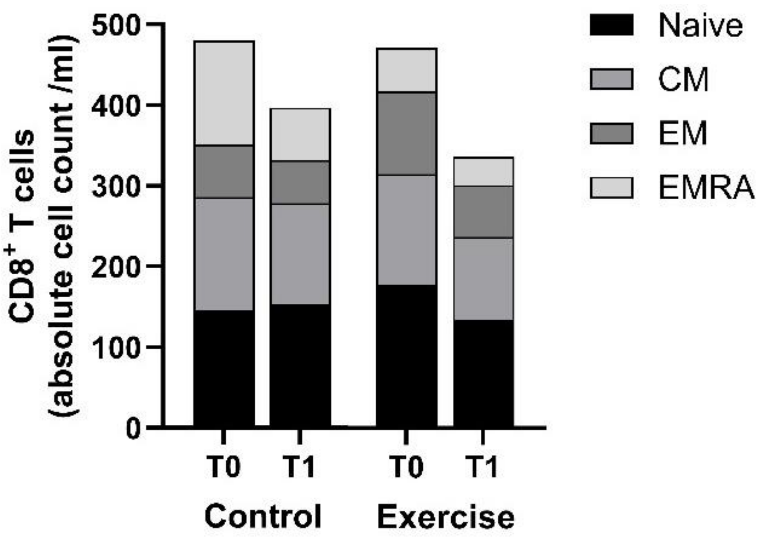

Supplementary Figure 4. Absolute counts of CD8^{+} T cell subsets. Absolute counts of the CD8^{+} T cell subsets per ml peripheral blood of patients with breast cancer from the exercise intervention (n=7) and control group (n=9) before (T0) and after (T1) six weeks of neoadjuvant chemotherapy. Linear regression analysis showed no significant between-group differences in the absolute cell counts of the CD8^{+} T cell subsets. Data is presented as mean values. Abbreviations: CM, central memory; EM, effector memory; EMRA, effector memory CD45RA^{+}.

A)

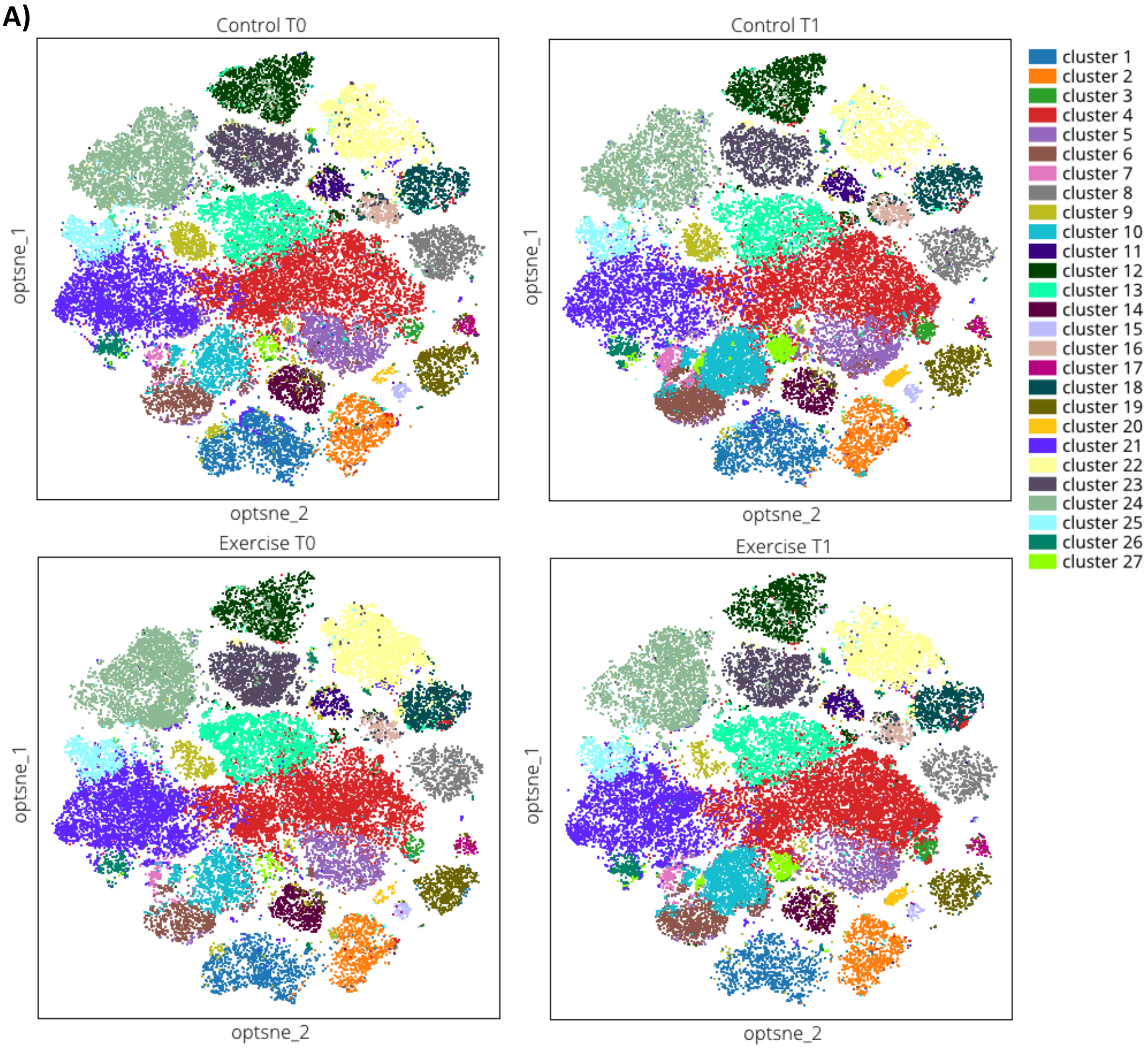

Supplementary Figure 5. Cluster analysis of NK cell phenotype. A) Cluster analysis of NK cells based on the expression of activating and inhibitory receptors in the exercise intervention (n=6) and control group (n=9) before (T0) and after (T1) six weeks of neoadjuvant chemotherapy. **B)** (next page) Heatmap showing the expression of each activating or inhibitory marker within each NK cell cluster. Red = high expression, blue = low expression.

B)

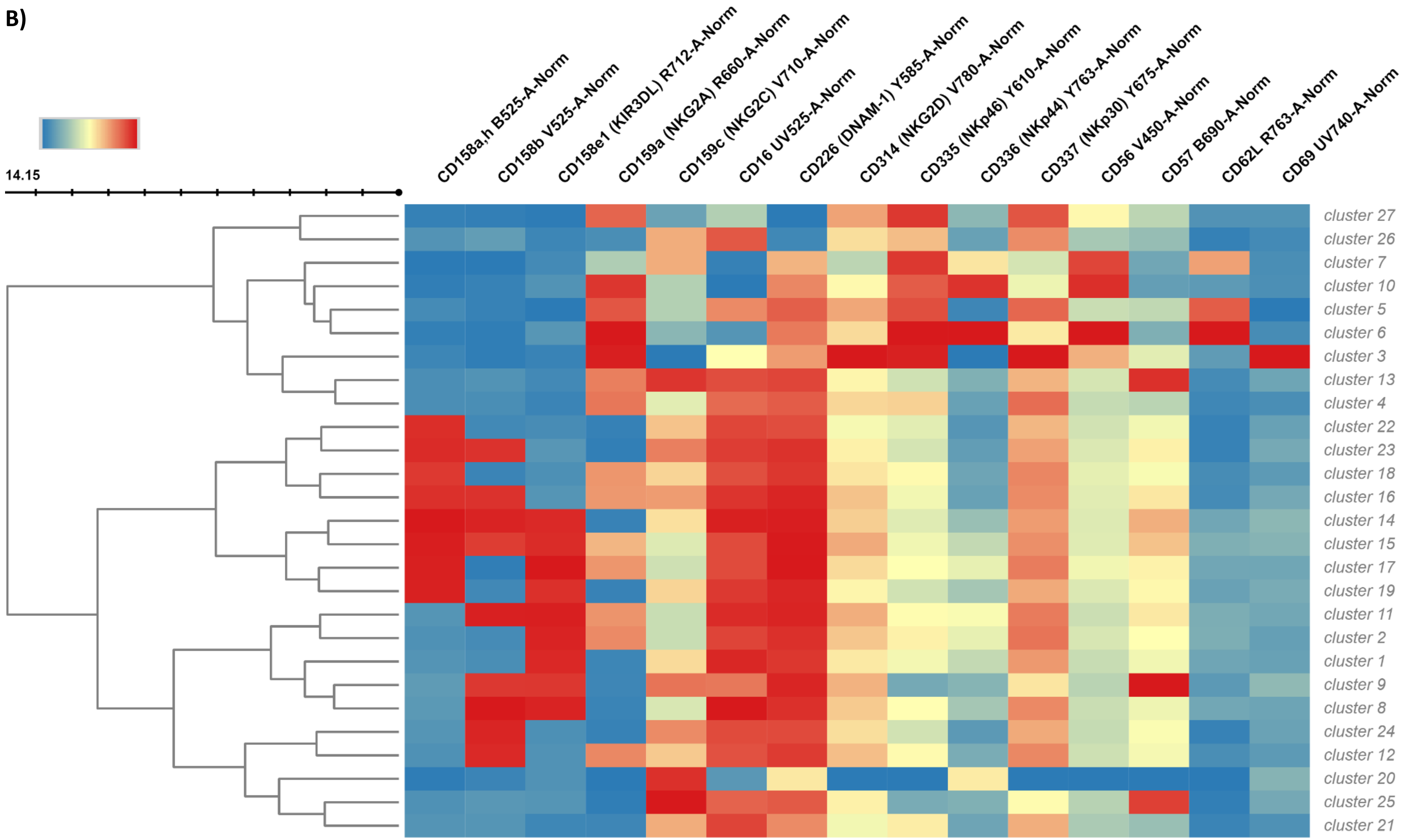
14.15
CD158a,h B525-A-Norm
CD158b V525-A-Norm
CD158e1 (KIR3DL) R712-A-Norm
CD159a (NKG2A) R660-A-Norm
CD159c (NKG2C) V710-A-Norm
CD16 UV525-A-Norm
CD226 (DNAM-1) Y585-A-Norm
CD314 (NKG2D) V780-A-Norm
CD335 (NKp46) Y610-A-Norm
CD336 (NKp44) Y763-A-Norm
CD337 (NKp30) Y675-A-Norm
CD56 V450-A-Norm
CD57 B690-A-Norm
CD62L R763-A-Norm
CD69 UV740-A-Norm
cluster 27
cluster 26
cluster 7
cluster 10
cluster 5
cluster 6
cluster 3
cluster 13
cluster 4
cluster 22
cluster 23
cluster 18
cluster 16
cluster 14
cluster 15
cluster 17
cluster 19
cluster 11
cluster 2
cluster 1
cluster 9
cluster 8
cluster 24
cluster 12
cluster 20
cluster 25
cluster 21

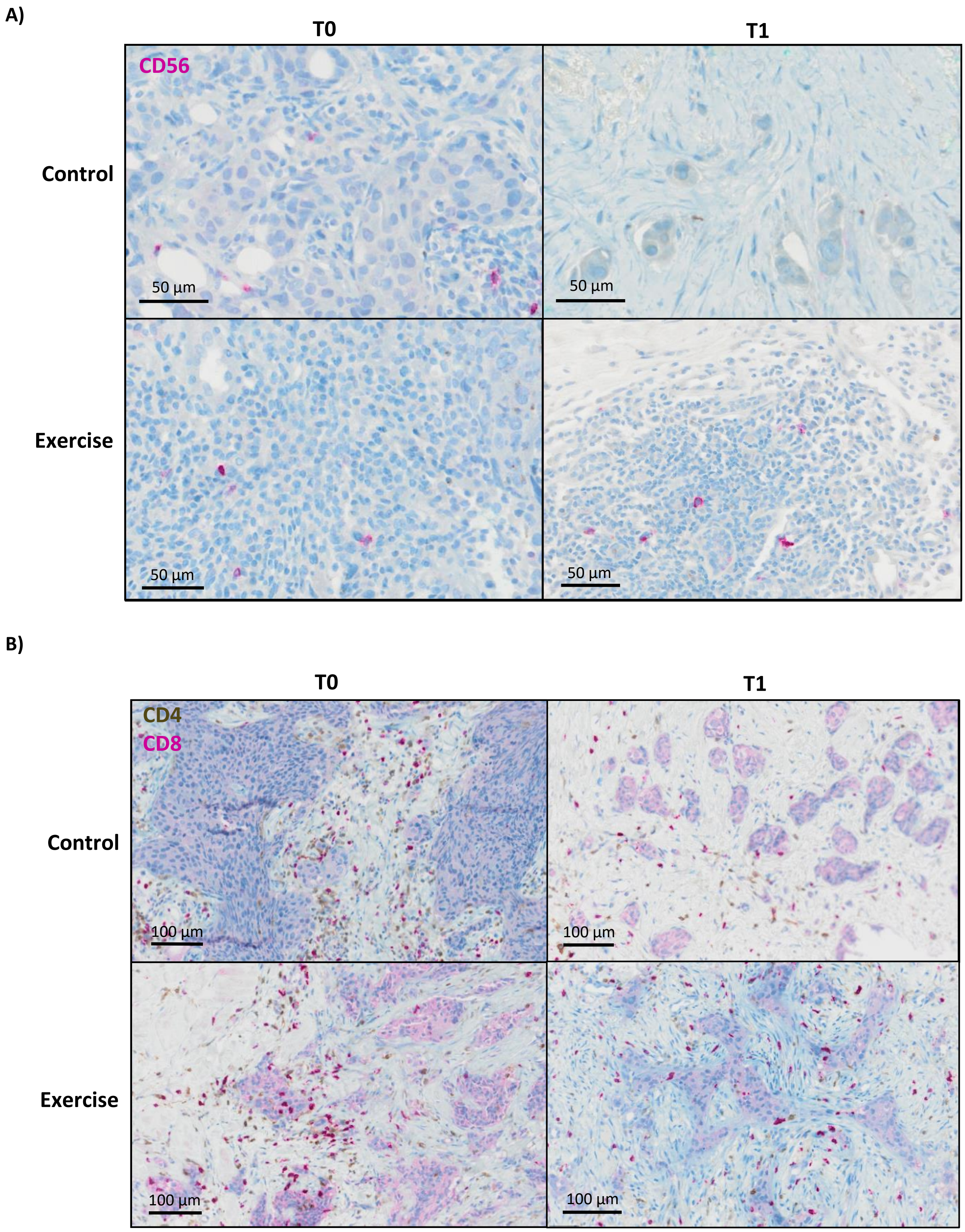

Supplementary Figure 6. Tumour infiltrating lymphocytes. The infiltration of **A)** CD56^{+} cells (red) and **B)** CD4^{+} (brown) and CD8^{+} (red) cells in tissue sections from biopsies of illustrative patients of the control group and the exercise group at T0 (baseline) and T1 (after six weeks of neoadjuvant chemotherapy).
