# Supplementary material for: Exploring the effects of exercise on immune cell function and tumour infiltration in patients with breast cancer receiving neoadjuvant chemotherapy – a feasibility trial
Source: Brain Behav Immun Health. 2025 May 20;46:101021. doi: 10.1016/j.bbih.2025.101021 (PMC12159211; doi:10.1016/j.bbih.2025.101021)
Supplement: Multimedia component 2 [file mmc2.pdf]

Supplementary Table 1. Flow cytometry panels immune cell profiling

Antigen	Clone	Fluorochrome	Company	Panel
CD14	M5E2	Pacific Blue	Miltenyi Biotech	2
CD16	B73.1	FITC	Biolegend	1
CD19	J3-119	ECD	Beckman Coulter	1
CD25	2A3	PE	BD Biosciences	2
CD27	1A4CD27	PC7	Beckman Coulter	2
CD3	BW264/56	VioGreen	Miltenyi Biotec	1
CD4	SK3	APC-H7	BD Biosciences	2
CD45	2D1	PerCP	BD Biosciences	1,2
CD45RA	2H4	ECD	Beckman Coulter	2
CD56	HCD56	BV421	Biolegend	1
CD8	B9.11	APC-AF700	Beckman Coulter	2

Supplementary Table 2. Flow cytometry panels NK cell phenotype

Antigen	Clone	Fluorochrome	Company	Panel
CD134 (OX40)	Ber-ACT35 (ACT35)	BV786	BD Biosciences	2
CD158a,h (KIR2DL1/DS1)	HP-MA4	FITC	BioLegend	1
CD158b (KIR2DL2/DL3)	CH-L	BV510	BD Biosciences	1
CD158e1 (KIR3DL1)	DX9	AF700	BioLegend	1
CD159a (NKG2A)	Z199	APC	Beckman Coulter	1
CD159c (NKG2C)	134591	BV711	BD Biosciences	1
CD16	3G8	BUV496	BD Biosciences	1,2
CD160	BY55	PE-Cy7	BioLegend	2
CD226 (DNAM-1) 11A8	11A8	BV510	BioLegend	2
CD226 (DNAM-1) 11A8	11A8	PE	BioLegend	1
CD244 (2B4)	C1.7	PerCP-Cy5.5	BioLegend	2
CD258 (LIGHT)	7-3 (7)	PE	eBioscience	2
CD27	O323	PE-Cy5	eBioscience	2
CD279 (PD-1)	EH12.2H7	BV711	BioLegend	2
CD3	UCHT1	BUV737	BD Biosciences	2
CD3	UCHT1	BV605	BioLegend	1
CD314 (NKG2D)	1D11	BV785	BioLegend	1
CD335 (NKp46)	9E2	PE-Dazzle594	BioLegend	1
CD336 (NKp44)	P44-8	PE-Cy7	BioLegend	1
CD337 (NKp30)	Z25	PE-Cy5	Beckman Coulter	1
CD366 (TIM-3)	F38-2E2	BV605	BioLegend	2
CD45	HI30	BUV395	BD Biosciences	1,2
CD56	HCD56	BV421	BioLegend	1,2
CD57	QA17A04	PerCP-Cy5.5	BioLegend	1
CD62L	DREG-56	APC-Fire750	BioLegend	1
CD69	FN50	BUV737	BD Biosciences	1
CD96	NK92.39	PE-Dazzle594	BioLegend	2
KLRG-1	REA261	FITC	Miltenyi Biotec	2
Live/dead	-	ViaKrome808	Beckman Coulter	1,2
SIGLEC7	6-434	APC-Fire750	BioLegend	2
SIGLEC9	191240	AF700	R&D systems	2
TIGIT	741182	APC	R&D systems	2

Supplementary Table 3. Flow cytometry panel NK cell function

Fluorochrome	Antigen	Clone	Company
APC	CD69	FN50	Biolegend
BV421	TNF*	MAb11	Biolegend
BV605	CD3	HIT3alpha	BD Biosciences
BV711	CD56	NCAM16.2	BD Biosciences
E780	Fixable viability dye	-	eBioscience
KO	CD45	J33	Beckman Coulter
PE-Cy7	IFN-y*	4S.B3	Invitrogen
PerCP-Cy5.5	Granzyme B*	QA16A02	Biolegend

** intracellular*

Supplementary Table 4. Immunohistochemistry protocols

Antigen	Clone	Company	Ventana Ultra Detection method*
CD4	SP35	ABCAM	Optiview detection kit (incl. DAB and CuSO4)
CD8	8/144B	DAKO	Ultraview Alkaline Phosphatase Red
Granzyme B	GB7	Monosan	Optiview detection kit (incl. DAB and CuSO4)
CD056	MRQ-42	Cellmarque	Ultraview Alkaline Phosphatase Red

*Tissue sections were pre-treated with CC1 for 24 minutes at 100°C for antigen retrieval, whereafter the primary antibody against granzyme B (Monosan, Uden, The Netherlands) or CD4 (Abcam, Cambridge, UK) was used in combination with the Optiview detection kit including DAB and CuSO4 (Roche, Basel, Switzerland) for detection and visualisation. Next, slides were treated again with CC1, and incubated with the primary antibody against CD56 (Cellmarque, Rocklin, USA) or CD8 (Dako, Glostrup, Denmark) which was detected with the Ultraview Alkaline Phosphatase Red. After immunohistochemical staining the sections were washed with EZ prep and soap, water, and dehydrated with baths of ethanol and cleared with xylene. All sections were mounted with Tissue Tek ® cover slipping film (Sakura Finetek Europe B.V., Alphen aan den Rijn, The Netherlands).

Supplementary Table 5. Tumour characteristics of patients with available biopsy data

	**Biopsy data (n=8)**	**No biopsy data (n=12)**
T-status, n (%)		
T1	0 (0%)	2 (17%)
T2	6 (75%)	4 (33%)
T3	2 (25%)	4 (33%)
T4	0 (0%)	2 (17%)
N+-status, n (%)		
N0	4 (50%)	7 (58%)
N1	3 (33%)	4 (33%)
N2	1 (17%)	1 (8%)
Tumour type, n (%)		
Infiltrating carcinoma NOS	6 (75%)	8 (67%)
Infiltrating lobular carcinoma	2 (25%)	4 (33%)
Breast cancer subtype, n (%)		
Triple negative	3 (38%)	5 (42%)
ER^+, PR^+, $Her2^-$	5 (63%)	7 (58%)

Abbreviations: NOS, not otherwise specified; ER, Estrogen Receptor; PR, Progesterone Receptor; Her2, Human Epidermal Growth Factor Receptor 2.

Supplementary Table 6. *Absolute counts or percentages (%) of immune cell subsets in peripheral blood of the exercise intervention (n=7) and control group (n=9) before (T0) and after (T1) 6 weeks of chemotherapy. The regression coefficients (β) indicate the between-group differences corrected for baseline values, as determined by linear regression analysis.*

**Variable**	***N***	**T0**	**T1**	**Between-Group change**	
		**Mean (SD)**	**Mean (SD)**	**β (95% CI)**	***p* value**
**Leukocytes (absolute cell count, x 10^6/ml)**					
Exercise	7	5.9 (1.9)	5.0 (4.5)	-1.8 (-5.9; 2.3)	0.36
Control	9	7.7 (3.4)	7.4 (2.6)		
**Lymphocytes (absolute cell count /ml)**					
Exercise	7	2062.7 (1146.6)	1038.9 (726.8)*	-236.3 (-782.7; 310.1)	0.37
Control	9	2184.0 (601.8)	1317.4 (417.5)*		
**% Monocytes of PBMCs**					
Exercise	7	11.9 (6.7)	15.2 (5.9)	-5.4 (-13.1; 2.4)	0.16
Control	9	11.2 (3.4)	20.1 (8.5)*		
**% B cells of lymphocytes**					
Exercise	7	10.4 (3.7)	1.1 (1.0)*	0.2 (-0.6; 1.1)	0.55
Control	9	10.7 (4.7)	0.9 (0.7)*		
**% NK cells of lymphocytes**					
Exercise	7	14.2 (6.2)	11.3 (7.8)	-0.3 (-6.1; 5.6)	0.92
Control	9	15.1 (5.0)	12.3 (6.2)		
**% $CD56^{dim}$ of NK cells**					
Exercise	7	96.3 (3.1)	93.9 (3.9)*	2.3 (-1.9; 6.5)	0.25
Control	9	96.4 (1.4)	91.8 (4.7)*		
**% $CD56^{bright}$ of NK cells**					
Exercise	7	3.7 (3.0)	6.0 (4.0)	-2.5 (-6.8; 1.8)	0.24
Control	9	3.4 (1.5)	8.2 (4.7)*		
**% NKT cells of lymphocytes**					
Exercise	7	4.4 (2.0)	9.9 (5.0)*	2.3 (-1.7; 6.4)	0.24
Control	9	7.2 (6.3)	11.0 (8.1)*		

**Variable**	***N***	**T0**	**T1**	**Between-Group change**	
		**Mean (SD)**	**Mean (SD)**	**β (95% CI)**	***p* value**
**% T cells of lymphocytes**					
Exercise	7	72.0 (6.7)	84.1 (8.2)*	1.2 (-5.2; 7.7)	0.69
Control	9	71.1 (6.5)	82.2 (7.3)*		
**% conventional $CD4^+$ T cells of T cells**					
Exercise	7	65.0 (3.3)	57.0 (6.4)*	-1.4 (-8.1; 5.3)	0.66
Control	9	61.2 (11.5)	54.9 (12.1)*		
% naïve of conventional $CD4^+$ T cells					
Exercise	7	50.2 (10.5)	35.0 (12.6)*	0.9 (-8.7; 10.5)	0.84
Control	9	54.8 (11.2)	40.1 (18.7)*		
% central memory of conventional $CD4^+$ T cells					
Exercise	7	39.0 (12.1)	48.6 (14.9)*	-0.2 (-7.3; 7.0)	0.96
Control	9	35.3 (9.3)	44.9 (10.9)*		
% effector memory of conventional $CD4^+$ T cells					
Exercise	7	9.1 (7.5)	13.8 (11.8)*	-0.9 (-2.5; 0.6)	0.22
Control	9	7.6 (4.6)	12.3 (8.5)*		
% EMRA of conventional $CD4^+$ T cells					
Exercise	7	1.8 (1.6)	2.6 (2.8)	0.5 (-0.9; 2.0)	0.43
Control	9	2.3 (3.0)	2.7 (4.2)		
**% Regulatory $CD4^+$ T cells of T cells**					
Exercise	7	6.9 (2.9)	6.8 (3.5)	-0.5 (-3.6; 2.7)	0.75
Control	9	5.3 (1.2)	6.0 (2.8)		
**% $CD8^+$ T cells of T cells**					
Exercise	7	30.7 (2.7)	38.0 (5.6)*	1.1 (-4.0; 6.2)	0.64
Control	9	31.9 (11.4)	38.1 (13.1)*		

Variable	*N*	T0	T1	Between-Group change	
		**Mean (SD)**	**Mean (SD)**	**β (95% CI)**	***p* value**
% naïve of CD8⁺ T cells					
Exercise	7	30.8 (19.8)	30.0 (17.3)	-5.0 (-9.4; -0.6)	**0.03**
Control	9	35.2 (19.6)	39.0 (19.4)[a]		
% central memory of CD8⁺ T cells					
Exercise	7	32.0 (13.1)	33.0 (14.6)	1.6 (-4.3; 7.5)	0.57
Control	9	30.6 (11.3)	30.2 (10.2)		
% effector memory of CD8⁺ T cells					
Exercise	7	24.0 (22.0)	25.7 (21.9)	3.3 (-1.4; 8.0)	0.15
Control	9	13.0 (7.6)	11.8 (7.3)		
% EMRA of CD8⁺ T cells					
Exercise	7	13.2 (9.9)	11.2 (8.5)	-0.7 (-5.1; 3.7)	0.75
Control	9	21.2 (24.3)	18.9 (22.0)*		

*Significant within-group difference between T0 and T1 based on Wilcoxon signed rank ($p<0.05$). [a]$p=0.05$.

Supplementary Table 7. *The %positive CD56^{dim} and CD56^{bright} NK cells for NK cell markers of the exercise intervention and control group before (T0) and after (T1) 6 weeks of chemotherapy. The regression coefficients (β) indicate the between-group differences corrected for baseline values, as determined by linear regression analysis.*

**Variable**	***N***	**T0**	**T1**	**Between-group change**	
		**Mean (SD)**	**Mean (SD)**	**β (95% CI)**	***p* value**
**%NKp30^{+} CD56^{dim} NK cells**					
Exercise	6	45.1 (10.0)	48.8 (9.5)	-1.0(-9.6; 7.7)	0.81
Control	9	41.3 (9.3)	46.4 (11.7)		
**%NKp44^{+} CD56^{dim} NK cells**					
Exercise	6	0.1 (0.1)	0.2 (0.1)	-0.0 (-0.2; 0.1)	0.54
Control	9	0.2 (0.4)	0.2 (0.2)		
**%NKp46^{+} CD56^{dim} NK cells**					
Exercise	6	76.7 (10.1)	77.8 (1.0)	1.0 (-6.4; 8.4)	0.78
Control	9	76.3 (8.8)	76.7 (8.0)		
**%NKG2C^{+} CD56^{dim} NK cells**					
Exercise	6	1.2 (0.8)	1.4 (1.0)	0.2 (-0.6; 1.0)	0.54
Control	9	1.9 (1.5)	1.9 (1.7)		
**%NKG2D^{+} CD56^{dim} NK cells**					
Exercise	6	91.2 (5.8)	84.5 (10.6)	-3.7 (-7.7; 0.3)	**0.06**
Control	9	94.2 (6.3)	92.0(6.5)*		
**%NKG2A^{+} CD56^{dim} NK cells**					
Exercise	6	45.2 (14.7)	52.2 (13.3)*	-1.8 (-11.3; 7.7)	0.69
Control	9	45.2 (15.1)	54.1 (12.3)*		
**%KIR2DL1/DS1^{+} CD56^{dim} NK cells**					
Exercise	6	26.1 (12.0)	22.4 (12.5)*	1.1 (-3.6; 5.9)	0.61
Control	9	22.6 (6.5)	18.1 (5.9)*		
**%KIR2DL2/DL3^{+} CD56^{dim} NK cells**					
Exercise	6	28.4 (12.9)	24.9 (10.4)	0.1 (-5.8; 6.0)	0.98
Control	9	34.4 (7.6)	28.6 (5.6)*		
**%KIR3DL1^{+} CD56^{dim} NK cells**					
Exercise	6	14.9 (17.6)	14.2 (17.3)	1.6 (-2.3; 5.4)	0.40
Control	9	19.6 (12.3)	17.0 (11.5)*		
**%CD57^{+} CD56^{dim} NK cells**					
Exercise	6	23.8 (9.5)	18.9 (7.9)*	-1.1 (-7.4; 5.3)	0.73
Control	9	27.2 (13.4)	22.6 (12.1)		

**Variable**	***N***	**T0**	**T1**	**Between-group change**	
		**Mean (SD)**	**Mean (SD)**	**β (95% CI)**	***p* value**
**%CD62L$^+$ CD56^{dim} NK cells**					
Exercise	6	8.4 (3.8)	8.4 (2.3)	-4.4 (-10.1; 1.4)	0.12
Control	9	12.6 (7.5)	13.5 (5.6)		
**%CD69$^+$ CD56^{dim} NK cells**					
Exercise	6	3.1 (2.8)	3.4 (1.6)	-0.3 (-1.7; 1.2)	0.69
Control	9	1.9 (0.9)	2.9 (1.6)*		
**%DNAM-1$^+$ CD56^{dim} NK cells**					
Exercise	6	94.1 (1.9)	90.9 (3.0)	3.0 (-1.6; 7.6)	0.18
Control	9	94.7 (3.7)	88.9 (8.3)*		
**%2B4$^+$ CD56^{dim} NK cells**					
Exercise	4	96.9 (1.9)	96.3 (1.6)	1.2 (-1.5; 4.0)	0.34
Control	9	96.2 (2.0)	94.5 (2.8)*		
**%LIGHT$^+$ CD56^{dim} NK cells**					
Exercise	4	0.5 (0.31)	0.6 (0.19)	-1.1 (-3.38; 1.23)	0.32
Control	9	1.0 (1.23)	1.7 (1.86)		
**%OX40$^+$ CD56^{dim} NK cells**					
Exercise	4	0.3 (0.2)	0.3 (0.2)	-0.0 (-0.6; 0.6)	0.93
Control	9	0.6 (0.8)	0.5 (0.7)		
**%CD160$^+$ CD56^{dim} NK cells**					
Exercise	4	31.7 (4.3)	21.4 (8.5)	-0.5 (-15.3; 14.2)	0.94
Control	9	45.0 (10.3)	28.2 (9.8)*		
**%KLRG-1$^+$ CD56^{dim} NK cells**					
Exercise	4	53.6 (15.9)	58.7 (13.9)	3.1 (-2.1; 8.3)	0.22
Control	9	51.2 (21.9)	53.5 (20.2)		
**%SIGLEC7$^+$ CD56^{dim} NK cells**					
Exercise	4	84.3 (6.2)	78.7 (10.3)	1.9 (-4.3; 8.2)	0.51
Control	9	90.0 (6.3)	83.0 (6.8)*		
**%SIGLEC9$^+$ CD56^{dim} NK cells**					
Exercise	4	0.4 (0.4)	0.8 (0.8)	-0.2 (-1.4; 1.0)	0.72
Control	9	0.5 (0.4)	1.1 (0.9)*		

**Variable**	***N***	**T0**	**T1**	**Between-group change**	
		**Mean (SD)**	**Mean (SD)**	**β (95% CI)**	***p* value**
**%TIGIT$^+$ CD56^{dim} NK cells**					
Exercise	4	46.8 (15.81)	51.6 (13.3)	1.4 (-3.3; 6.1)	0.53
Control	9	51.6 (10.30)	54.3 (9.6)		
**%CD96$^+$ CD56^{dim} NK cells**					
Exercise	4	26.2 (9.8)	26.5 (4.1)	-8.2 (-16.8; 0.5)	**0.06**
Control	9	32.2 (11.0)	35.3 (6.6)		
**%TIM-3$^+$ CD56^{dim} NK cells**					
Exercise	4	0.2 (0.2)	0.1 (0.1)	-0.2 (-0.5; 0.2)	0.29
Control	9	0.3 (0.2)	0.4 (0.4)		
**%PD-1$^+$ CD56^{dim} NK cells**					
Exercise	4	0.2 (0.1)	0.3 (0.2)	0.0 (-0.3; 0.3)	0.98
Control	9	0.4 (0.2)	0.5 (0.3)		
**%CD27$^+$ CD56^{dim} NK cells**					
Exercise	4	4.5 (1.0)	5.6 (1.9)	-2.5 (-7.2; 2.3)	0.28
Control	9	4.8 (1.5)	8.3 (3.9)*		
**%NKp30$^+$ CD56^{bright} NK cells**					
Exercise	6	18.3 (5.8)	22.8 (5.0)	2.4 (-4.2; 9.0)	0.45
Control	9	19.8 (7.6)	21.3 (7.9)		
**%NKp44$^+$ CD56^{bright} NK cells**					
Exercise	6	3.7 (1.5)	6.2 (3.3)*	-0.0 (-2.7; 2.6)	0.97
Control	9	2.2 (1.0)	4.4 (1.5)*		
**%NKp46$^+$ CD56^{bright} NK cells**					
Exercise	6	97.0 (2.2)	97.2 (1.8)	-0.5 (-2.1; 1.1)	0.53
Control	9	98.3 (0.7)	98.1 (0.9)		
**%NKG2C$^+$ CD56^{bright} NK cells**					
Exercise	6	4.5 (5.3)	2.4 (2.4)	-0.2 (-1.4; 1.0)	0.71
Control	9	4.9 (1.8)	2.8 (1.6)*		
**%NKG2D$^+$ CD56^{bright} NK cells**					
Exercise	6	91.1 (9.8)	89.9 (9.4)	-2.5 (-5.6; 0.7)	0.11
Control	9	88.7 (10.2)	90.4 (8.0)		

Variable	*N*	T0	T1	Between-group change	
		Mean (SD)	Mean (SD)	β (95% CI)	*p* value
**%NKG2A$^+$ CD56^{bright} NK cells**					
Exercise	6	91.4 (4.7)	92.1 (6.4)	0.2 (-2.5; 2.9)	0.85
Control	9	93.3 (3.2)	94.0 (3.3)		
**%KIR2DL1/DS1$^+$ CD56^{bright} NK cells**					
Exercise	6	3.0 (2.6)	2.2 (1.3)	0.1 (-0.9; 1.1)	0.84
Control	9	2.3 (1.2)	1.7 (1.2)		
**%KIR2DL2/DL3$^+$ CD56^{bright} NK cells**					
Exercise	6	3.5 (1.9)	3.1 (1.2)	-0.0 (-1.0; 0.9)	0.94
Control	9	4.6 (1.3)	3.7 (1.0)*		
**%KIR3DL1$^+$ CD56^{bright} NK cells**					
Exercise	6	2.2 (2.6)	2.0 (2.0)	0.3 (-0.8; 1.4)	0.55
Control	9	2.9 (1.6)	2.3 (1.6)		
**%CD57$^+$ CD56^{bright} NK cells**					
Exercise	6	1.4 (1.0)	1.4 (1.2)	0.3 (-0.8; 1.4)	0.59
Control	9	0.8 (0.5)	1.0 (0.6)		
**%CD62L$^+$ CD56^{bright} NK cells**					
Exercise	6	45.4 (14.2)	34.9 (8.4)	-9.5 (-26.8; 7.7)	0.25
Control	9	48.3 (21.1)	44.2 (17.2)		
**%CD69$^+$ CD56^{bright} NK cells**					
Exercise	6	3.5 (1.4)	3.8 (0.9)	0.6 (-0.9; 2.0)	0.41
Control	9	2.4 (1.0)	2.7 (1.4)		
**%DNAM-1$^+$ CD56^{bright} NK cells**					
Exercise	6	94.7 (1.9)	89.6 (7.1)*	0.6 (-4.7; 5.9)	0.82
Control	9	92.5 (6.5)	84.9 (12.7)*		
**%2B4$^+$ CD56^{bright} NK cells**					
Exercise	4	87.8 (8.4)	88.9 (2.6)	2.2 (-2.6; 7.1)	0.33
Control	9	82.3 (6.6)	83.0 (6.5)		
**%LIGHT$^+$ CD56^{bright} NK cells**					
Exercise	4	2.1 (1.1)	2.7 (1.6)	-0.0 (-2.0; 1.9)	0.97
Control	9	2.5 (2.8)	2.7 (1.3)		

**Variable**	***N***	**T0**	**T1**	**Between-group change**	
		**Mean (SD)**	**Mean (SD)**	**β (95% CI)**	***p* value**
**%OX40^{+} CD56^{bright} NK cells**					
Exercise	4	0.2 (0.3)	0.3 (0.3)	0.1 (-0.7; 0.8)	0.85
Control	9	0.5 (0.7)	0.4 (0.8)		
**%CD160^{+} CD56^{bright} NK cells**					
Exercise	4	6.7 (4.2)	7.0 (2.5)	2.0 (-0.9; 4.8)	0.16
Control	9	6.6 (2.7)	5.0 (2.3)		
**%KLRG-1^{+} CD56^{bright} NK cells**					
Exercise	4	8.3 (4.9)	9.6 (4.6)	2.2 (-2.4; 6.8)	0.32
Control	9	10.8 (9.4)	9.6 (8.9)		
**%SIGLEC7^{+} CD56^{bright} NK cells**					
Exercise	4	65.7 (8.7)	62.6 (10.6)	0.9 (-7.5; 9.4)	0.81
Control	9	70.6 (5.3)	66.2 (6.9)*		
**%SIGLEC9^{+} CD56^{bright} NK cells**					
Exercise	4	0.1 (0.1)	0.1 (0.1)	-0.1 (-0.3; 0.1)	0.16
Control	9	0.1 (0.1)	0.2 (0.1)		
**%TIGIT^{+} CD56^{bright} NK cells**					
Exercise	4	23.5 (9.9)	20.8 (4.9)	3.3 (-2.3; 8.9)	0.22
Control	9	20.5 (5.1)	15.7 (5.6)*		
**%CD96^{+} CD56^{bright} NK cells**					
Exercise	4	89.9 (4.2)	90.4 (3.2)	-0.8 (-7.6; 6.0)	0.79
Control	9	84.7 (6.1)	88.5 (5.8)		
**%TIM-3^{+} CD56^{bright} NK cells**					
Exercise	4	0.4 (0.5)	0.2 (0.4)	-0.1 (-0.6; 0.4)	0.77
Control	9	0.5 (0.5)	0.4 (0.5)		
**%PD-1^{+} CD56^{bright} NK cells**					
Exercise	4	0.9 (0.8)	0.8 (0.9)	0.3 (-0.3; 0.8)	0.27
Control	9	0.9 (0.8)	0.5 (0.4)		
**%CD27^{+} CD56^{bright} NK cells**					
Exercise	4	53.1 (15.4)	41.3 (21.2)	2.7 (-9.2; 14.6)	0.62
Control	9	62.0 (9.5)	48.7 (12.4)*		

*Significant within-group difference between T0 and T1 based on Wilcoxon signed rank ($p<0.05$).

Supplementary Table 8. *The percentage positive NK cells or the median fluorescence intensity (MFI) of the total or positive NK cell population after 4h co-culture with K562 tumour cells before (T0) and after (T1) 6 weeks of chemotherapy. The regression coefficients (β) indicate the between-group differences corrected for baseline values.*

**Variable**	***N***	**T0**	**T1**	**Between-group change**	
		**Mean (SD)**	**Mean (SD)**	**β (95% CI)**	***p* value**
**%CD107a$^+$ NK cells**					
Exercise	7	10.2 (3.6)	6.7 (2.1)*	0.80(-2.6; 4.2)	0.62
Control	9	14.3 (7.3)	8.2 (5.4)*		
**MFI CD107a$^+$ NK cells**					
Exercise	7	3067.8 (1440.7)	3623.9 (1563.7)	1038.5 (56.9; 2020.2)	**0.04**
Control	9	2280.7 (639.3)	2065.0 (410.9)		
MFI CD107a$^+$ CD56^{dim} NK cells					
Exercise	7	3138.6 (1543.7)	4013.2 (2089.4)	1482.1 (-55.0; 3019.1)	**0.06**
Control	9	2282.5 (646.7)	2067.1 (396.7)		
MFI CD107a$^+$ CD56^{bright} NK cells					
Exercise	7	2291.7 (1107.6)	3498.8 (3058.9)	300.9 (-3129.2; 3731.1)	0.85
Control	9	2271.0 (1518.0)	3362.6 (1963.2)		
**%CD69$^+$ NK cells**					
Exercise	7	80.3 (12.9)	71.3 (15.0)	12.5 (-4.8; 29.7)	0.14
Control	9	85.6 (9.5)	65.4 (23.7)*		
**MFI CD69$^+$ NK cells**					
Exercise	7	3115.6 (1609.7)	2291.8 (1324.0)	-99.5 (-1650.9; 1451.8)	0.89
Control	9	4466.1 (1923.8)	2590.4 (1286.2)*		
**%Granzyme$^+$ NK cells**					
Exercise	7	94.6 (3.0)	93.2 (3.3)	0.0 (-4.2; 4.3)	0.98
Control	9	92.3 (5.6)	92.4 (4.4)		
**MFI Granzyme$^+$ NK cells**					
Exercise	7	7867.7 (2794.3)	8228.4 (2770.9_	435.6 (-1150.0; 2021.3)	0.56
Control	9	7675.4 (1955.9)	7642.7 (1893.3)		
**%IFN-γ$^+$ NK cells**					
Exercise	7	0.3 (0.1)	0.1 (0.1)*	-0.3 (-0.8; 0.1)	0.15
Control	9	0.5 (0.3)	0.4 (0.5)		
**MFI IFN-γ total NK cells**					
Exercise	7	127.5 (35.6)	117.5 (32.6)*	-0.0 (-16.3; 16.3)	1.00
Control	9	140.1 (24.0)	127.5 (22.8)		

**Variable**	***N***	**T0**	**T1**	**Between-group change**	
		**Mean (SD)**	**Mean (SD)**	**β (95% CI)**	***p* value**
**%TNF⁺ NK cells**					
Exercise	7	0.4 (0.1)	0.3 (0.2)	-0.1 (-0.4; 0.1)	0.30
Control	9	0.6 (0.4)	0.5 (0.3)		
**MFI TNF total NK cells**					
Exercise	7	243.7 (46.1)	359.4 (86.8)*	-1.1 (-81.8; 79.7)	0.98
Control	9	265.3 (52.1)	383.7 (88.3)*		
**%K562 lysis**					
Exercise	7	-26.6 (30.1)	-20.3 (18.2)	18.8 (-3.9; 41.5)	**0.10**
Control	9	-36.4 (37.7)	-45.2 (35.8)		

*Significant within-group difference between T0 and T1 based on Wilcoxon signed rank ($p<0.05$).

Supplementary Table 9. *Percentage positive NK cells or the median fluorescence intensity (MFI) of the total or positive NK cell population after overnight stimulation with IL2/IL15 and subsequent 4h co-culture with K562 tumour cells before (T0) and after (T1) six weeks of chemotherapy.*

**Variable**	***N***	**T0**	**T1**	**Between-group change**	
		**Mean (SD)**	**Mean (SD)**	**β (95% CI)**	***p* value**
**%CD107a$^+$ NK cells IL2/IL15**					
Exercise	7	48.3 (4.8)	44.6 (6.4)	-4.1 (-10.5; 2.3)	0.19
Control	9	51.8 (5.6)	50.4 (5.5)		
**MFI CD107a$^+$ NK cells IL2/IL15**					
Exercise	7	3327.5 (1003.9)	3095.7 (892.1)	198.2 (-465.6; 862.0)	0.53
Control	9	3288.9 (799.1)	2872.3 (774.7)		
**%CD69$^+$ NK cells IL2/IL15**					
Exercise	7	99.6 (0.2)	99.2 (0.4)*	-0.10 (-0.74; 0.54)	0.75
Control	9	99.5 (0.3)	99.3 (0.7)		
**MFI CD69$^+$ NK cells IL2/IL15**					
Exercise	7	9025.1 (1881.8)	6295.3 (1155.0)*	-524.5 (-2495.8; 1446.8)	0.58
Control	9	9744.8 (993.4)	7053.9 (2089.0)*		
**%Granzyme $^+$ NK cells IL2/IL15**					
Exercise	7	99.5 (0.6)	99.4 (0.4)	0.0 (-0.4; 0.5)	0.84
Control	9	99.5 (0.7)	99.3 (0.5)		
**MFI Granzyme$^+$ NK cells IL2/IL15**					
Exercise	7	15093.0 (3789.0)	16267.2 (3126.3)	-1396.1 (-5255.0; 2462.7)	0.45
Control	9	18373.6 (5898.5)	19340.4 (4781.6)		
**%IFN-γ $^+$ NK cells IL2/IL15**					
Exercise	7	8.0 (4.0)	6.0 (2.8)	-2.1 (-6.7; 2.6)	0.35
Control	9	13.9 (7.4)	9.3 (4.6)		
**MFI IFN-γ total NK cells IL2/IL15**					
Exercise	7	316.0 (116.5)	252.6 (96.1)*	-33.6 (-128.7; 61.5)	0.46
Control	9	407.1 (143.2)	317.0 (86.8)		
**%TNF $^+$ NK cells IL2/IL15**					
Exercise	7	6.6 (3.0)	6.0 (2.6)	-2.5 (-6.3; 1.2)	0.17
Control	9	7.2 (2.4)	9.0 (4.7)		
**MFI TNF total NK cells IL2/IL15**					
Exercise	7	313.1 (67.7)	414.9 (109.1)*	-13.4 (-114.7; 87.9)	0.78
Control	9	328.0 (68.5)	439.3 (96.8)*		

**Variable**	***N***	**T0**	**T1**	**Between-group change**	
		**Mean (SD)**	**Mean (SD)**	**β (95% CI)**	***p* value**
**%K562 lysis IL2/IL15**					
Exercise	7	43.5 (32.6)	15.1 (38.3)*	-0.5 (-28.9; 28.0)	0.97
Control	9	39.9 (29.9)	13.3 (26.1)*		

*Significant within-group difference between T0 and T1 based on Wilcoxon signed rank (p<0.05).
